# 6-[(4′-Ethoxycarbonyl-[1,1′-biphenyl]-4-yl)oxy]hexanoic acid

**DOI:** 10.1107/S1600536813025877

**Published:** 2013-09-25

**Authors:** Delia López-Velázquez, Angel Mendoza

**Affiliations:** a Facultad de Ciencias Químicas, Benemérita Universidad Autónoma de Puebla, Puebla, Pue, Mexico; b Centro de Química, ICUAP, Benemérita Universidad Autónoma de Puebla, Puebla, Pue, Mexico

## Abstract

In the title compound, C_21_H_24_O_5_, the dihedral angle between the benzene rings is 19.57 (15)°. In the crystal, the mol­ecular arrangement makes up head-to-head centrosymmetric dimers assembled by pairs of O—H⋯O bonds; this arrangement builds a graph-set ring motif of *R* ^2^_2_(8). The dimers are linked into a tape running along the *b-*axis direction through C—H⋯O inter­actions. The packing is further consolidated by C—H⋯π inter­actions, forming layers parallel to (10-2).

## Related literature

For hydrogen-bonding assemblies, see: Braga *et al.* (2004 ▶). For hydrogen-bonding packing modes and applications of hydrogen bonds, see: Jeong *et al.* (2006 ▶); Leiserowitz (1976 ▶). For graph-set analysis of hydrogen bonds, see: Etter *et al.* (1990 ▶); Bernstein *et al.* (1995 ▶).
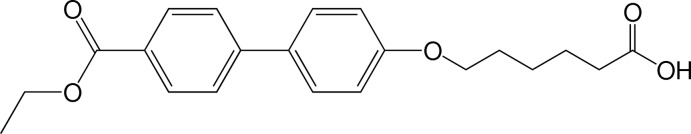


## Experimental

### 

#### Crystal data

C_21_H_24_O_5_
*M* *_r_* = 356.4
Monoclinic, 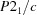

*a* = 9.111 (2) Å
*b* = 14.753 (3) Å
*c* = 14.427 (2) Å
β = 100.785 (14)°
*V* = 1904.9 (6) Å^3^
*Z* = 4
Mo *K*α radiation
μ = 0.09 mm^−1^
*T* = 298 K
0.5 × 0.5 × 0.4 mm

#### Data collection

Siemens P4 diffractometer
Absorption correction: ψ scan (North *et al.*, 1968 ▶) *T*_min_ = 0.891, *T*_max_ = 0.911
4265 measured reflections
3217 independent reflections
1628 reflections with *I* > 2σ(*I*)
*R*_int_ = 0.031
3 standard reflections every 97 reflections intensity decay: 6%

#### Refinement

*R*[*F* ^2^ > 2σ(*F* ^2^)] = 0.059
*wR*(*F* ^2^) = 0.171
*S* = 1.06
3217 reflections
238 parameters
H-atom parameters constrained
Δρ_max_ = 0.16 e Å^−3^
Δρ_min_ = −0.15 e Å^−3^

### 

Data collection: *XSCANS* (Siemens, 1994 ▶); cell refinement: *XSCANS*; data reduction: *XSCANS*; program(s) used to solve structure: *SHELXS2013* (Sheldrick, 2008 ▶); program(s) used to refine structure: *SHELXL2013* (Sheldrick, 2008 ▶); molecular graphics: *ORTEP-3 for Windows* (Farrugia, 2012 ▶); software used to prepare material for publication: *WinGX* (Farrugia, 2012 ▶).

## Supplementary Material

Crystal structure: contains datablock(s) global, I. DOI: 10.1107/S1600536813025877/is5302sup1.cif

Structure factors: contains datablock(s) I. DOI: 10.1107/S1600536813025877/is5302Isup2.hkl

Additional supplementary materials:  crystallographic information; 3D view; checkCIF report

## Figures and Tables

**Table 1:** Hydrogen-bond geometry (Å, °) *Cg*1 is the centroid of the C13–C18 ring.

*D*—H⋯*A*	*D*—H	H⋯*A*	*D*⋯*A*	*D*—H⋯*A*
O1—H1⋯O2^i^	0.82	1.80	2.612 (3)	170
C18—H18⋯O1^ii^	0.93	2.54	3.460 (4)	173
C6—H6*A*⋯*Cg*1^iii^	0.97	2.78	3.668 (4)	152

Symmetry codes: (i) 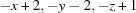; (ii) 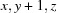; (iii) 
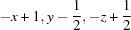
.

**Fig. 1.**
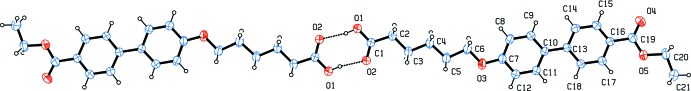
A view of the centrosymmetric dimer of the title compound. Displacement ellipsoids are drawn at the 50% probability level.

**Fig. 2.**
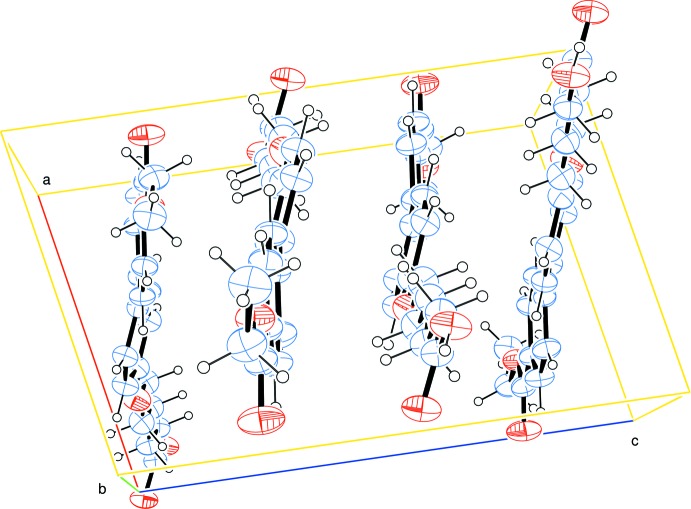
A crystal packing view of the title compound. Displacement ellipsoids are drawn at the 50% probability level.

## supplementary crystallographic information

### 1. Comment

Hydrogen bonds are the strongest of the non-covalent interactions and have a high degree of directionality. Therefore, the design of molecules with hydrogen bonding capabilities is very important due to its numerous potential application (Braga *et al.*, 2004) in nanotechnology, in crystal engineering, in template synthesis of polymers and networks, as well as in templated processes in biology such as the replication and transcription of nucleic acids. It has long been known that monocarboxylic acid may be interlinked to form the cyclic hydrogen-bonded dimer. This kind of molecular dimer, is well known as supramolecular synthon in crystals of carboxylic acids (Jeong *et al.*, 2006; Leiserowitz *et al.*, 1976). It is also important to point out that the title compound I contains a polymerizable end-group. Therefore it is a precursor for polymeric materials.

In the title compound, the ASU shows a molecule with two non-coplanar phenyl rings bonded by C10 and C13, both rings with *p*-substitution. The dihedral angle between these planes is 19.57 (15)°. On the other hand, C2 to C6 show an aliphatic extended-chain probably due to intermolecular interactions. The crystal packing makes up a head to head dimer assembled by intermolecular O—H···O bonds between the carboxyl groups (Fig. 1). This arrangement builds a graph-set ring *R*^2^_2_(8) (Etter *et al.*, 1990; Bernstein *et al.*, 1995). Two more interactions C—H···O and C—H···π interactions, are identified, which stabilize the crystal packing. A tape of molecules from the C18—H18···O1 interaction is formed along the *b* axis. The C6—H6A···*Cg*1 interaction is building a layer of molecules parallel to (1 0 2). (Table 1; *Cg*1 is the centroid of the ring composed of C13–C18.)

### 2. Experimental

6.2 g (14 mmol) of benzyl 6-(ethyl 4'-oxydiphenyl-4-carboxylate)-hexanoate was added to 90 ml of dry ethyl acetate. 5% Pd—C (0.029 g) was then added with stirring. The hydrogenolisis was allowed to proceed for 8.5 h under hydrogen atmosphere at room temperature. After the removal of the catalyst by filtration and evaporation of the ethyl acetate under reduced pressure, the residue was then dissolved in hot methylene chloride and the solution was allowed to cool to -10 °C. It gave a white crystalline solid which was filtered off (4.8 g, 13.48 mmol, yield 97%). Crystals of I were grown from a solution of acetone by slow evaporation technique at room temperature. Anal. Calc. for C_21_H_24_O_5_: C 70.79, H 6.74%. Found: C 70.86, H 6.92%. IR(solid state, cm^-1^): ν (C—H_Ar_) 3028; ν (C—H_Aliph_) 2938; ν (C= O, Aliph.) 1703; ν (C=O, COOH) 1694; ν (C=C, Ar) 1600. ^1^H NMR [400 MHz; CDCl_3_, (CH_3_)_4_Si) *δ* (p.p.m.)]: 1.40 (t, 3H_21_, CH_3_), 1.55 (m, 2H_4_, CH_2_), 1.74 (m, 2H_3_, CH_2_), 1.84 (m, 2H_5_, CH_2_), 2.41 (t, 2H_2_, CH_2_—COOH), 4.01 (t, 2H_6_, O—CH_2_), 4.38 (q, 2H_20_ Me—CH_2_—O), 6.98 (d, 2H), 7.55 (d, 2H), 7.62 (d, 2H), 8.07 (d, 2H). ^13^C NMR [100 MHz; CDCl_3_, (CH_3_)_4_Si *δ* (p.p.m.)]: C_21_ 14.61, C_4_ 24.64, C_3_ 25.82, C_5_, 29.15, C_2_ 34.05, C_20_ 61.15, C_6_ 67.93, C_12_ and C_8_ 115.11, C_15_ and C_17_ 126.63, C_14_ and C_18_ 128.57, C_9_ and C_11_ 130.29, C_10_ 132.57, C_16_ 145.36, C_13_ 145.36, C_7_ 159.48, C_19_ 166.87, C_1_ 179.41.

### 3. Refinement

H atoms linked to C and O atoms were placed in geometrical idealized positions (C—H = 0.93–0.97 Å and O—H = 0.82 Å) and refined as riding on their parent atoms, with *U*_iso_(H) = 1.2 *U*_eq_(C) or 1.5 *U*_eq_(C_methyl_, O).

### Crystal data

**Table:**

C_21_H_24_O_5_	*F*(000) = 760
*M**_r_* = 356.4	*D*_x_ = 1.243 Mg m^−^^3^
Monoclinic, *P*2_1_/*c*	Mo *K*α radiation, λ = 0.71073 Å
Hall symbol: -P 2ybc	Cell parameters from 43 reflections
*a* = 9.111 (2) Å	θ = 9.1–33.6°
*b* = 14.753 (3) Å	µ = 0.09 mm^−^^1^
*c* = 14.427 (2) Å	*T* = 298 K
β = 100.785 (14)°	PRISM, colorless
*V* = 1904.9 (6) Å^3^	0.5 × 0.5 × 0.4 mm
*Z* = 4	

### Data collection

**Table:**

Siemens P4 diffractometer	*R*_int_ = 0.031
Graphite monochromator	θ_max_ = 24.7°, θ_min_ = 2.0°
ω scans	*h* = −1→10
Absorption correction: ψ scan (North *et al.*, 1968)	*k* = −17→1
*T*_min_ = 0.891, *T*_max_ = 0.911	*l* = −16→16
4265 measured reflections	3 standard reflections every 97 reflections
3217 independent reflections	intensity decay: 6%
1628 reflections with *I* > 2σ(*I*)	

### Refinement

**Table:**

Refinement on *F*^2^	Hydrogen site location: inferred from neighbouring sites
Least-squares matrix: full	H-atom parameters constrained
*R*[*F*^2^ > 2σ(*F*^2^)] = 0.059	*w* = 1/[σ^2^(*F*_o_^2^) + (0.0433*P*)^2^ + 0.9974*P*] where *P* = (*F*_o_^2^ + 2*F*_c_^2^)/3
*wR*(*F*^2^) = 0.171	(Δ/σ)_max_ < 0.001
*S* = 1.06	Δρ_max_ = 0.16 e Å^−^^3^
3217 reflections	Δρ_min_ = −0.15 e Å^−^^3^
238 parameters	Extinction correction: *SHELXL*
0 restraints	Extinction coefficient: 0.0013 (4)
Primary atom site location: structure-invariant direct methods	

### Special details

**Table:**

Geometry. All e.s.d.'s (except the e.s.d. in the dihedral angle between two l.s. planes) are estimated using the full covariance matrix. The cell e.s.d.'s are taken into account individually in the estimation of e.s.d.'s in distances, angles and torsion angles; correlations between e.s.d.'s in cell parameters are only used when they are defined by crystal symmetry. An approximate (isotropic) treatment of cell e.s.d.'s is used for estimating e.s.d.'s involving l.s. planes.

### Fractional atomic coordinates and isotropic or equivalent isotropic displacement parameters (Å^2^)

**Table:**

	*x*	*y*	*z*	*U*_iso_*/*U*_eq_	
C1	0.8791 (4)	−0.8969 (2)	0.4560 (2)	0.0645 (9)	
C2	0.7761 (4)	−0.82076 (19)	0.4252 (2)	0.0672 (9)	
H2A	0.6847	−0.8309	0.4491	0.081*	
H2B	0.7504	−0.8216	0.3569	0.081*	
C3	0.8346 (4)	−0.72759 (19)	0.4559 (3)	0.0712 (10)	
H3A	0.9243	−0.7157	0.4309	0.085*	
H3B	0.8608	−0.7256	0.5242	0.085*	
C4	0.7197 (4)	−0.65502 (19)	0.4220 (2)	0.0721 (10)	
H4A	0.6936	−0.6578	0.3538	0.087*	
H4B	0.6301	−0.6678	0.4469	0.087*	
C5	0.7716 (4)	−0.5605 (2)	0.4506 (3)	0.0790 (11)	
H5A	0.8617	−0.5473	0.4265	0.095*	
H5B	0.7956	−0.5568	0.5189	0.095*	
C6	0.6529 (4)	−0.4906 (2)	0.4133 (3)	0.0748 (10)	
H6A	0.6288	−0.4933	0.345	0.09*	
H6B	0.5626	−0.5026	0.4378	0.09*	
C7	0.6196 (4)	−0.3305 (2)	0.4188 (2)	0.0666 (9)	
C8	0.4757 (4)	−0.3335 (2)	0.3664 (2)	0.0688 (9)	
H8	0.4334	−0.3885	0.3442	0.083*	
C9	0.3949 (4)	−0.2531 (2)	0.3471 (2)	0.0639 (9)	
H9	0.2982	−0.2558	0.3121	0.077*	
C10	0.4537 (3)	−0.16963 (19)	0.3782 (2)	0.0550 (8)	
C11	0.5982 (4)	−0.1695 (2)	0.4320 (2)	0.0655 (9)	
H11	0.6409	−0.1148	0.4551	0.079*	
C12	0.6788 (4)	−0.2480 (2)	0.4516 (2)	0.0706 (10)	
H12	0.7747	−0.2455	0.4876	0.085*	
C13	0.3703 (3)	−0.08361 (19)	0.3554 (2)	0.0560 (8)	
C14	0.2155 (4)	−0.0809 (2)	0.3260 (2)	0.0724 (10)	
H14	0.1615	−0.1347	0.3219	0.087*	
C15	0.1407 (4)	−0.0002 (2)	0.3031 (3)	0.0750 (10)	
H15	0.0375	−0.0008	0.2835	0.09*	
C16	0.2158 (3)	0.0809 (2)	0.3086 (2)	0.0598 (8)	
C17	0.3689 (4)	0.0802 (2)	0.3380 (2)	0.0657 (9)	
H17	0.4218	0.1345	0.3424	0.079*	
C18	0.4447 (3)	−0.0007 (2)	0.3611 (2)	0.0631 (9)	
H18	0.5479	0.0004	0.3809	0.076*	
C19	0.1335 (4)	0.1663 (2)	0.2828 (2)	0.0699 (9)	
C20	0.1574 (4)	0.3263 (2)	0.2690 (3)	0.0835 (11)	
H20A	0.0745	0.3366	0.3012	0.1*	
H20B	0.1206	0.3301	0.2016	0.1*	
C21	0.2767 (4)	0.3949 (2)	0.2988 (3)	0.0951 (13)	
H21A	0.3595	0.3827	0.2681	0.143*	
H21B	0.3095	0.392	0.366	0.143*	
H21C	0.2381	0.4544	0.2815	0.143*	
O1	0.8276 (3)	−0.97632 (15)	0.42837 (19)	0.0836 (8)	
H1	0.8874	−1.0153	0.4517	0.125*	
O2	1.0048 (3)	−0.88595 (14)	0.50571 (18)	0.0784 (7)	
O3	0.7090 (3)	−0.40416 (14)	0.44254 (17)	0.0856 (8)	
O4	−0.0006 (3)	0.17132 (17)	0.2554 (2)	0.1023 (9)	
O5	0.2228 (2)	0.23813 (15)	0.29348 (17)	0.0774 (7)	

### Atomic displacement parameters (Å^2^)

**Table:**

	*U*^11^	*U*^22^	*U*^33^	*U*^12^	*U*^13^	*U*^23^
C1	0.060 (2)	0.0473 (19)	0.085 (2)	0.0006 (17)	0.0096 (18)	0.0006 (18)
C2	0.068 (2)	0.0511 (18)	0.081 (2)	0.0086 (17)	0.0084 (18)	0.0084 (17)
C3	0.071 (2)	0.0500 (19)	0.093 (3)	0.0087 (17)	0.0146 (19)	0.0075 (18)
C4	0.085 (2)	0.0500 (19)	0.079 (2)	0.0125 (18)	0.0099 (19)	0.0045 (17)
C5	0.095 (3)	0.0496 (19)	0.089 (3)	0.0114 (19)	0.010 (2)	0.0040 (18)
C6	0.090 (3)	0.0483 (19)	0.086 (3)	0.0049 (18)	0.014 (2)	0.0031 (18)
C7	0.074 (2)	0.0485 (19)	0.075 (2)	0.0104 (18)	0.0078 (18)	−0.0006 (17)
C8	0.078 (2)	0.0464 (18)	0.079 (2)	0.0005 (17)	0.0067 (19)	−0.0051 (17)
C9	0.0571 (19)	0.0562 (19)	0.075 (2)	0.0030 (16)	0.0026 (16)	−0.0032 (17)
C10	0.0584 (19)	0.0447 (17)	0.0610 (19)	0.0020 (15)	0.0088 (15)	−0.0036 (14)
C11	0.066 (2)	0.0474 (18)	0.078 (2)	0.0013 (16)	0.0001 (17)	−0.0009 (16)
C12	0.064 (2)	0.054 (2)	0.086 (3)	0.0002 (17)	−0.0035 (18)	0.0006 (18)
C13	0.0538 (19)	0.0515 (18)	0.062 (2)	0.0040 (15)	0.0105 (15)	−0.0023 (15)
C14	0.056 (2)	0.058 (2)	0.102 (3)	−0.0038 (17)	0.0136 (19)	−0.0019 (19)
C15	0.0468 (19)	0.068 (2)	0.109 (3)	0.0068 (18)	0.0090 (19)	0.000 (2)
C16	0.0518 (19)	0.0542 (19)	0.073 (2)	0.0053 (15)	0.0115 (16)	−0.0018 (16)
C17	0.057 (2)	0.0541 (19)	0.082 (2)	0.0047 (16)	0.0026 (17)	−0.0025 (17)
C18	0.0499 (18)	0.0530 (19)	0.083 (2)	0.0054 (16)	0.0033 (16)	−0.0031 (17)
C19	0.059 (2)	0.066 (2)	0.084 (3)	0.0098 (19)	0.0146 (19)	0.0019 (19)
C20	0.079 (2)	0.061 (2)	0.109 (3)	0.019 (2)	0.016 (2)	0.017 (2)
C21	0.099 (3)	0.063 (2)	0.118 (3)	0.004 (2)	0.008 (3)	0.010 (2)
O1	0.0658 (15)	0.0533 (14)	0.121 (2)	0.0020 (12)	−0.0096 (14)	−0.0037 (14)
O2	0.0587 (14)	0.0538 (14)	0.1133 (19)	0.0005 (11)	−0.0082 (13)	−0.0025 (13)
O3	0.0923 (18)	0.0503 (13)	0.1049 (19)	0.0138 (13)	−0.0050 (15)	−0.0019 (13)
O4	0.0565 (15)	0.0853 (19)	0.160 (3)	0.0175 (14)	0.0074 (16)	0.0118 (18)
O5	0.0668 (15)	0.0565 (14)	0.1050 (19)	0.0122 (12)	0.0063 (13)	0.0084 (13)

### Geometric parameters (Å, º)

**Table:**

C1—O2	1.243 (4)	C10—C13	1.484 (4)
C1—O1	1.296 (3)	C11—C12	1.371 (4)
C1—C2	1.478 (4)	C11—H11	0.93
C2—C3	1.511 (4)	C12—H12	0.93
C2—H2A	0.97	C13—C18	1.393 (4)
C2—H2B	0.97	C13—C14	1.395 (4)
C3—C4	1.513 (4)	C14—C15	1.381 (4)
C3—H3A	0.97	C14—H14	0.93
C3—H3B	0.97	C15—C16	1.373 (4)
C4—C5	1.505 (4)	C15—H15	0.93
C4—H4A	0.97	C16—C17	1.380 (4)
C4—H4B	0.97	C16—C19	1.478 (4)
C5—C6	1.519 (4)	C17—C18	1.388 (4)
C5—H5A	0.97	C17—H17	0.93
C5—H5B	0.97	C18—H18	0.93
C6—O3	1.408 (4)	C19—O4	1.214 (4)
C6—H6A	0.97	C19—O5	1.327 (4)
C6—H6B	0.97	C20—O5	1.446 (4)
C7—O3	1.364 (3)	C20—C21	1.489 (5)
C7—C12	1.378 (4)	C20—H20A	0.97
C7—C8	1.386 (4)	C20—H20B	0.97
C8—C9	1.396 (4)	C21—H21A	0.96
C8—H8	0.93	C21—H21B	0.96
C9—C10	1.384 (4)	C21—H21C	0.96
C9—H9	0.93	O1—H1	0.82
C10—C11	1.398 (4)		
			
O2—C1—O1	122.4 (3)	C11—C10—C13	120.8 (3)
O2—C1—C2	122.6 (3)	C12—C11—C10	121.7 (3)
O1—C1—C2	114.9 (3)	C12—C11—H11	119.1
C1—C2—C3	115.7 (3)	C10—C11—H11	119.1
C1—C2—H2A	108.3	C11—C12—C7	120.9 (3)
C3—C2—H2A	108.3	C11—C12—H12	119.5
C1—C2—H2B	108.3	C7—C12—H12	119.5
C3—C2—H2B	108.3	C18—C13—C14	116.5 (3)
H2A—C2—H2B	107.4	C18—C13—C10	120.9 (3)
C2—C3—C4	111.4 (3)	C14—C13—C10	122.6 (3)
C2—C3—H3A	109.4	C15—C14—C13	121.6 (3)
C4—C3—H3A	109.4	C15—C14—H14	119.2
C2—C3—H3B	109.4	C13—C14—H14	119.2
C4—C3—H3B	109.4	C16—C15—C14	121.3 (3)
H3A—C3—H3B	108	C16—C15—H15	119.4
C5—C4—C3	113.9 (3)	C14—C15—H15	119.4
C5—C4—H4A	108.8	C15—C16—C17	118.4 (3)
C3—C4—H4A	108.8	C15—C16—C19	120.3 (3)
C5—C4—H4B	108.8	C17—C16—C19	121.3 (3)
C3—C4—H4B	108.8	C16—C17—C18	120.6 (3)
H4A—C4—H4B	107.7	C16—C17—H17	119.7
C4—C5—C6	111.5 (3)	C18—C17—H17	119.7
C4—C5—H5A	109.3	C17—C18—C13	121.7 (3)
C6—C5—H5A	109.3	C17—C18—H18	119.1
C4—C5—H5B	109.3	C13—C18—H18	119.1
C6—C5—H5B	109.3	O4—C19—O5	123.2 (3)
H5A—C5—H5B	108	O4—C19—C16	124.5 (3)
O3—C6—C5	108.3 (3)	O5—C19—C16	112.4 (3)
O3—C6—H6A	110	O5—C20—C21	107.2 (3)
C5—C6—H6A	110	O5—C20—H20A	110.3
O3—C6—H6B	110	C21—C20—H20A	110.3
C5—C6—H6B	110	O5—C20—H20B	110.3
H6A—C6—H6B	108.4	C21—C20—H20B	110.3
O3—C7—C12	116.1 (3)	H20A—C20—H20B	108.5
O3—C7—C8	124.8 (3)	C20—C21—H21A	109.5
C12—C7—C8	119.0 (3)	C20—C21—H21B	109.5
C7—C8—C9	119.4 (3)	H21A—C21—H21B	109.5
C7—C8—H8	120.3	C20—C21—H21C	109.5
C9—C8—H8	120.3	H21A—C21—H21C	109.5
C10—C9—C8	122.2 (3)	H21B—C21—H21C	109.5
C10—C9—H9	118.9	C1—O1—H1	109.5
C8—C9—H9	118.9	C7—O3—C6	118.7 (3)
C9—C10—C11	116.6 (3)	C19—O5—C20	118.3 (3)
C9—C10—C13	122.5 (3)		
			
O2—C1—C2—C3	1.4 (5)	C18—C13—C14—C15	0.6 (5)
O1—C1—C2—C3	−179.4 (3)	C10—C13—C14—C15	−178.5 (3)
C1—C2—C3—C4	−179.1 (3)	C13—C14—C15—C16	−0.3 (6)
C2—C3—C4—C5	179.9 (3)	C14—C15—C16—C17	−0.1 (5)
C3—C4—C5—C6	179.1 (3)	C14—C15—C16—C19	179.4 (3)
C4—C5—C6—O3	−179.9 (3)	C15—C16—C17—C18	0.2 (5)
O3—C7—C8—C9	179.7 (3)	C19—C16—C17—C18	−179.3 (3)
C12—C7—C8—C9	0.7 (5)	C16—C17—C18—C13	0.2 (5)
C7—C8—C9—C10	0.3 (5)	C14—C13—C18—C17	−0.5 (5)
C8—C9—C10—C11	−1.2 (5)	C10—C13—C18—C17	178.6 (3)
C8—C9—C10—C13	178.0 (3)	C15—C16—C19—O4	−0.4 (6)
C9—C10—C11—C12	1.1 (5)	C17—C16—C19—O4	179.1 (4)
C13—C10—C11—C12	−178.1 (3)	C15—C16—C19—O5	179.1 (3)
C10—C11—C12—C7	−0.1 (5)	C17—C16—C19—O5	−1.4 (5)
O3—C7—C12—C11	−179.9 (3)	C12—C7—O3—C6	178.2 (3)
C8—C7—C12—C11	−0.9 (5)	C8—C7—O3—C6	−0.8 (5)
C9—C10—C13—C18	−159.7 (3)	C5—C6—O3—C7	−178.5 (3)
C11—C10—C13—C18	19.4 (5)	O4—C19—O5—C20	−1.6 (5)
C9—C10—C13—C14	19.3 (5)	C16—C19—O5—C20	178.9 (3)
C11—C10—C13—C14	−161.5 (3)	C21—C20—O5—C19	173.0 (3)

### Hydrogen-bond geometry (Å, º)

*Cg*1 is the centroid of the C13–C18 ring.

**Table:**

*D*—H···*A*	*D*—H	H···*A*	*D*···*A*	*D*—H···*A*
O1—H1···O2^i^	0.82	1.80	2.612 (3)	170
C18—H18···O1^ii^	0.93	2.54	3.460 (4)	173
C6—H6*A*···*Cg*1^iii^	0.97	2.78	3.668 (4)	152

Symmetry codes: (i) −*x*+2, −*y*−2, −*z*+1; (ii) *x*, *y*+1, *z*; (iii) −*x*+1, *y*−1/2, −*z*+1/2.
